# SAGE: a comprehensive resource of genetic variants integrating South Asian whole genomes and exomes

**DOI:** 10.1093/database/bay080

**Published:** 2018-09-13

**Authors:** Judith Mary Hariprakash, Shamsudheen Karuthedath Vellarikkal, Ankit Verma, Anop Singh Ranawat, Rijith Jayarajan, Rowmika Ravi, Anoop Kumar, Vishal Dixit, Ambily Sivadas, Atul Kumar Kashyap, Vigneshwar Senthivel, Paras Sehgal, Vijayalakshmi Mahadevan, Vinod Scaria, Sridhar Sivasubbu

**Affiliations:** 1 GN Ramachandran Knowledge Center for Genome Informatics, Council of Scientific and Industrial Research (CSIR) Institute of Genomics & Integrative Biology, Mathura Road, Delhi 110025, India; 2 Genomics & Molecular Medicine, Council of Scientific and Industrial Research (CSIR) Institute of Genomics & Integrative Biology, Mathura Road, Delhi 110025, India; 3 School of Chemical & Biotechnology, Shanmugha Arts, Science, Technology and Research Academy (SASTRA) University, Thanjavur, Tamil Nadu 613402, India

## Abstract

South Asia is home to $\sim $20% of the world population and characterized by distinct ethnic, linguistic, cultural and genetic lineages. Only limited representative samples from the region have found its place in large population-scale international genome projects. The recent availability of genome scale data from multiple populations and datasets from South Asian countries in public domain motivated us to integrate the data into a comprehensive resource. In the present study, we have integrated a total of six datasets encompassing 1213 human exomes and genomes to create a compendium of 154 814 557 genetic variants and adding a total of 69 059 255 novel variants. The variants were systematically annotated using public resources and along with the allele frequencies are available as a browsable-online resource South Asian genomes and exomes. As a proof of principle application of the data and resource for genetic epidemiology, we have analyzed the pathogenic genetic variants causing retinitis pigmentosa. Our analysis reveals the genetic landscape of the disease and suggests subset of genetic variants to be highly prevalent in South Asia.

## Introduction

South Asia is home to more than 20% of the total world population (1, 2) and characterized by distinct ethnic, cultural and linguistic differences and genetic diversity (1). The demographic and genetic diversity in this region has also been significantly shaped by human migration (1). High genetic differentiation and structure of Asian population have also been attributed to various factors like antiquity, founder effects, endogamy (3) and consanguinity. In addition, Asian population has been expanding rapidly in the recent past, resulting in a large number of polymorphic sites in the genome (4, 5).

South Asia has been highlighted as a region having one of the highest levels of genetic diversity after Africa (6–8). Though densely and diversely populated, South Asia has been underrepresented in global genome studies (6). This lacuna makes it especially challenging for genetic epidemiology as well as to map, prioritize and understand disease-causing mutations in Mendelian diseases until the recent past (9). In addition, population-scale genomic maps would also serve as a starting point for imputation as well as fine mapping of functional variants for complex diseases. The availability of a genetic landscape of the region would undoubtedly provide the much required baseline data for genetic epidemiology as evident from recent studies on Middle Eastern population genomes (10–12). Such a resource would undoubtedly be the starting point towards enabling precision medicine.

The advent and rapid adoption of next generation sequencing has seen a number of genome and population-scale initiatives emerge trying to address specific population groups and varied questions varying from ancestry and admixture to disease predisposition and epidemiology (13). A number of initiatives in this space are worth mentioning. One of the key projects that have reported the genetic diversity of the Asian subcontinent is the Human Genome Organization Pan-Asian Single Nucleotide Polymorphism (SNP) consortium (version 16) (14), which relied on genotyping arrays to define the population architecture. Phases II and III of the 1000 genome project (1KGP) (15) have sequenced individuals from South Asian ancestry as well. The Exome Variant Server (16), created as part of the National Heart, Lung, and Blood Institute (NHLBI) Exome Sequencing Project (ESP) 1, includes frequency information from South and East Asians. Various personal genomes from this region have also been sequenced, e.g. Kitzman *et al.* (17), Patowary *et al.* (18) and Gupta *et al.* (19). The decreasing costs of whole-genome sequencing have made large deep-sequencing initiatives such as Singapore Sequencing Indian Project (SSIP) (20) and the South Asian genome (SAG) (21) possible.

Motivated by the availability of whole genome and whole exome sequences from multiple projects, we have created a comprehensive resource for South Asian human genetic variation, integrating datasets from the region. As a case study highlighting the utility of such a resource, we have analyzed potential pathogenic variants of retinitis pigmentosa (RP) based on allele frequencies. The global prevalence of RP is around 1 in 4000 (22), while it has been reported in some Asian populations to be nearly 1 in 1000 (23, 24). Our analysis reveals genetic variants associated with RP occur at higher frequencies in South Asians compared to global populations and suggests prevalent mutations, which has implications in the diagnosis of patients.

This database, South Asian Genomes and Exomes (SAGE), will serve as a starting point for population-specific genomic medicine and thus would enable and accelerate translational genomics in the region. The resource is publicly available at the URL http://clingen.igib.res.in/SAGE.

## Materials and Methods

### Datasets

The whole genome and whole exome datasets originating from South Asian populations available in the public domain were systematically compiled. This compilation encompassed a total of six datasets namely SAG (21), South Asian samples (SAS) from 1000 genome consortium data (15), SSIP (20), Wellness genome project (WGP), population genetics of Andamanese (PGA) (25) and SIMONS genome diversity project (SGDP) (26). The SAG dataset consists of 174 whole genome and 146 whole exome sequence variants contributing to a total of 321 samples. SAS from 1000 genomes consortium data encompasses a total of 661 genomes. SSIP dataset comprises whole genome data of 36 individuals of South Indian ancestry from Singapore. WGP consists of 93 samples with 30 whole genomes and 63 exomes from our in-house data of healthy Indians with a median age of 93. PGA consists of 79 genomes of which 10 individuals are from the Jarawa and Onge populations in the Andaman Islands and 69 genomes representing rest of India. SGDP consists of 300 individuals from 142 populations, from which we have obtained 39 individuals with South Asian ancestry. All the datasets were based on the Human genome 19 (GRCh37/hg19) assembly. The variants were retrieved in variant call format from individual datasets and integrated into a master compendium using bespoke scripts in Python.

### Annotation of variants

Annotation of variants was performed using Annotate Variation (ANNOVAR) (27) across a number of public databases and computational tools. Locations of the variant with respect to the genic boundaries were obtained from RefSeq database. The functional consequences of an amino acid change as a result of exonic variants were also obtained from the RefSeq database. An array of computational tools including SIFT, Polyphen, MutationTaster, MutationAccessor, FATHMM, RadialSVM, CADD, LR and phylop were used to annotate the effect or functionality of variants. The pathogenicity of the variants were retrieved from another popular database, ClinVar to annotate the variants as pathogenic, likely pathogenic, benign and likely benign. In addition, allelic frequency of the variants were also obtained from other global population datasets including ExAC 0.3 (28), 1000 genomes 2015aug (15) and esp6500si (16). Variants that were previously not reported in dbSNP142, ExAC 0.3 or the 1000 genomes 2015aug datasets were classified as novel variants.

### Allele and genotype frequency calculations

The variant call files were analyzed using bespoke python script to calculate the allele and genotype frequencies. Minor allele frequencies (MAFs) (a) for each population were calculated as follows:

$$ f(a)= \frac{Aa+2\times (aa)}{2\times (AA)+2\times (Aa)+2\times (aa.)}$$

While the genotype frequency (aa) was calculated as follows:

$$ f(aa)=\frac{aa}{AA+ Aa+ aa} $$

### Database and web server

The variant data as well as the annotation were formatted to JavaScript Object Notation and ported onto MongoDB 3.4.1 (29). The web interface for querying the database was written in PERL/CGI and JavaScript. The web server was configured in Apache 2.4.12.

### Population stratification analysis

Genotype data for 2500 individuals in the 1KGP encompassing over 84 801 880 autosomal SNPs were used for the analysis. This dataset represents 22 populations across four major geographical regions (America, Europe, Africa and East Asia). The 1000 genome dataset was merged with SSIP, SAG, SGDP, WGP, SAS and PGA datasets. The data were further filtered for individual and genotype missingness >5%. Variants were further filtered for MAF <5% and a Hardy–Weinberg equilibrium (HWE) deviation with *P* < 5x10^−6^. This resulted in a total of 26 720 shared SNPs. Multidimensional scale (MDS) analysis was performed using PLINK v1.9 (https://www.cog-genomics.org/plink2). The plots were visualized using rgl (29) package in R.

### Variants associated with RP and annotation

The variants associated with RP were retrieved from ClinVar (June 2017), a comprehensive online resource of clinically significant genetic variants. We obtained 427 SNPs belonging to 45 genes relating to RP. All single nucleotide variants used were tested for HWE in the study population. Observed genotype frequencies were compared with those expected under HWE using the $\chi$^2^ test. South Asian allele frequencies were compared with that of the 1KGP (Phase 3) global population average (ALL) using Fisher’s exact test. The distribution of allele frequencies among various populations were visualized by a scatter plot in Python’s matplotlib package (30).

## Results

### Compendium of genetic variants

We collated a compendium of 154 814 557 genetic variants from six datasets from South Asia.

The variant compilation was derived from datasets of 1213 individuals sequenced as part of different studies and available in public domain. This included studies like SAG that sequenced 321 South Asians to smaller projects like the PGA, which involved 79 whole genomes of Andamanese. The distribution of samples, genomes, exomes and variants are summarized in Table 1.

**Table 1:** Summary of the Asian whole genome and whole exome data aggregated from various publications and sources

**Project**	**Pop. code**	**Cohort size**	**Whole genomes**	**Whole exomes**	**Description of the samples**	**No. of SNVs**	**No. of InDels**	**Ref.**
	PJL	108	108		Punjabi from Lahore, Pakistan			
	STU	111	111		Sri Lankan Tamil from UK			
**1000G SAS**	GIH	108	108		Gujarati Indian from Houston	84 801 880	36 000 000	(15)
	BEB	105	105		Bengali from Bangladesh			
	ITU	112	112		Indian Telugu from UK			
**The SAG**	SAG	321	174	147	SAG	11 624 872	1 352 706	(21)
**Indians in Singapore**	SSIP	36	36	0	Indians living in Singapore	10 286 478	1 269 000	(20)
**Wellness Genomics Project**	WGP	93	30	63	Genomes of the centenarians	46 256 520	2 520 066	In-house data
**PGA**	PGA	79	79	0	Andaman genomes	13 679 600	1 574 694	(25)
**SGDP**	SGDP	39	39	0	SIMON Genomes	34 400 000	2 100 000	(26)
**Total**		**1213**	**902**	**210**				

Systematic comparison of these variants revealed that 69 059 255 variants as unique and not found in any of the global sequencing datasets including 1000 genomes, ExAC or dbSNP.

### Classification of genetic variants

**Figure 1:**
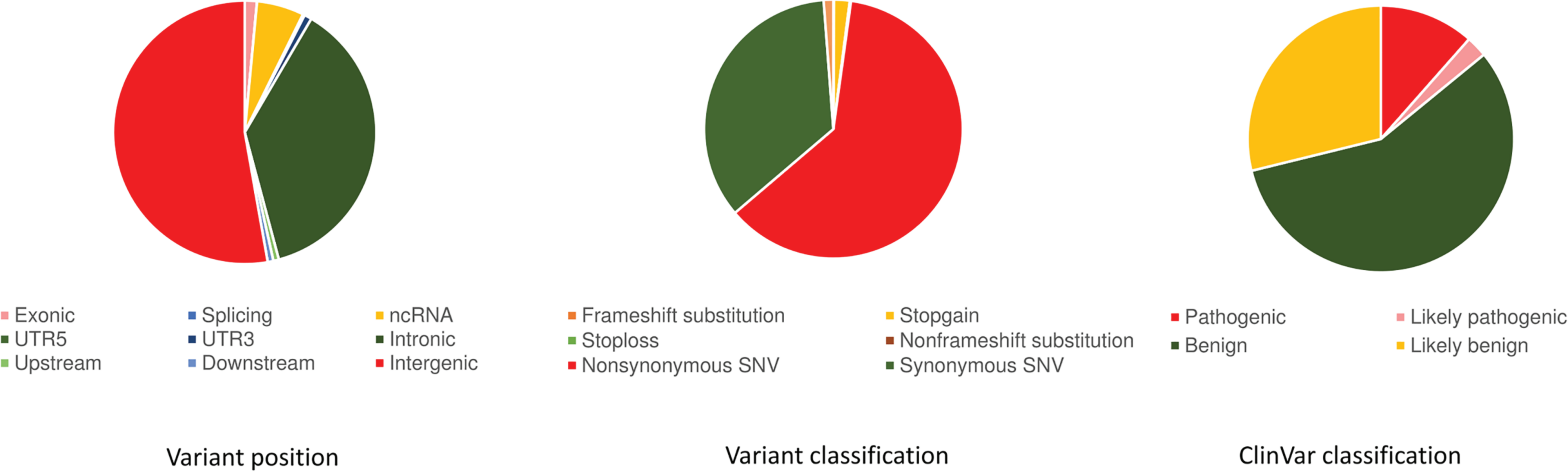
Variant annotation. (a) Gene-based annotation: variant annotation revealing the position of the variants in the genomic region. (b) Functional consequences of the variant. (c) Filter-based annotation: clinical significance of variant based on ClinVar classification.

The compendium of genetic variants was systematically annotated using a number of databases as well as computational tools using ANNOVAR. Briefly, the variants were annotated based on the context of a gene, the genomic loci and the functionality. Analysis of the genomic variants with respect to protein-coding RefSeq genes revealed a total of 2 248 780 variants (1.49%) as exonic, 56 453 145 (37.37%) as intronic and 24 263 (0.01%) were at the splice sites. Of the total number of variants, 499 323 variants (0.33%) mapped to to ncRNA exons, 8 256 022 variants (5.46%) mapped to introns of ncRNAs and 3065 variants (0.002%) mapped to splice sites of ncRNA. Distribution of variants in terms of gene boundaries is depicted in Figure 1a.

Further annotation of the exonic variants in protein-coding genes suggested 1 077 570 variants were non-synonymous and a total of 612 466 were synonymous variants. Similarly, a total of 34 319 variants resulted in a potential gain of a stop codon (stop gain) and 1530 variants resulted in a potential loss of a stop codon (stop loss) depicted in Figure 1b.

The ClinVar database was used to annotate variants of clinical significance. Out of 154 million genetic variants, a total of 31 027 variants had annotations in ClinVar of which 2366 variants were annotated as pathogenic and 527 were annotated as likely pathogenic, 11 720 as benign and 5920 as likely benign variants (Figure 1c). ClinVar annotated 38 variants as drug response that can potentially affect the therapeutic response of at least 13 different drugs.

Additional classification of variants was attempted using computational tools. A total of 904 689 variants were annotated as pathogenic by an array of nine computational tools. The summary of annotations for individual computational methods is available at **Supplementary Table 1**.

### Population stratification analysis

To examine the population affinities of the samples included in the present analysis, we performed an MDS of the variants with the prominent global populations derived from the 1KGP as described earlier. For this, we considered the shared genotypes between 1023 South Asians and 2014 samples from 1KGP representing 22 populations across four major geographical regions (America, Africa, Europe and East Asia).

**Figure 2:**
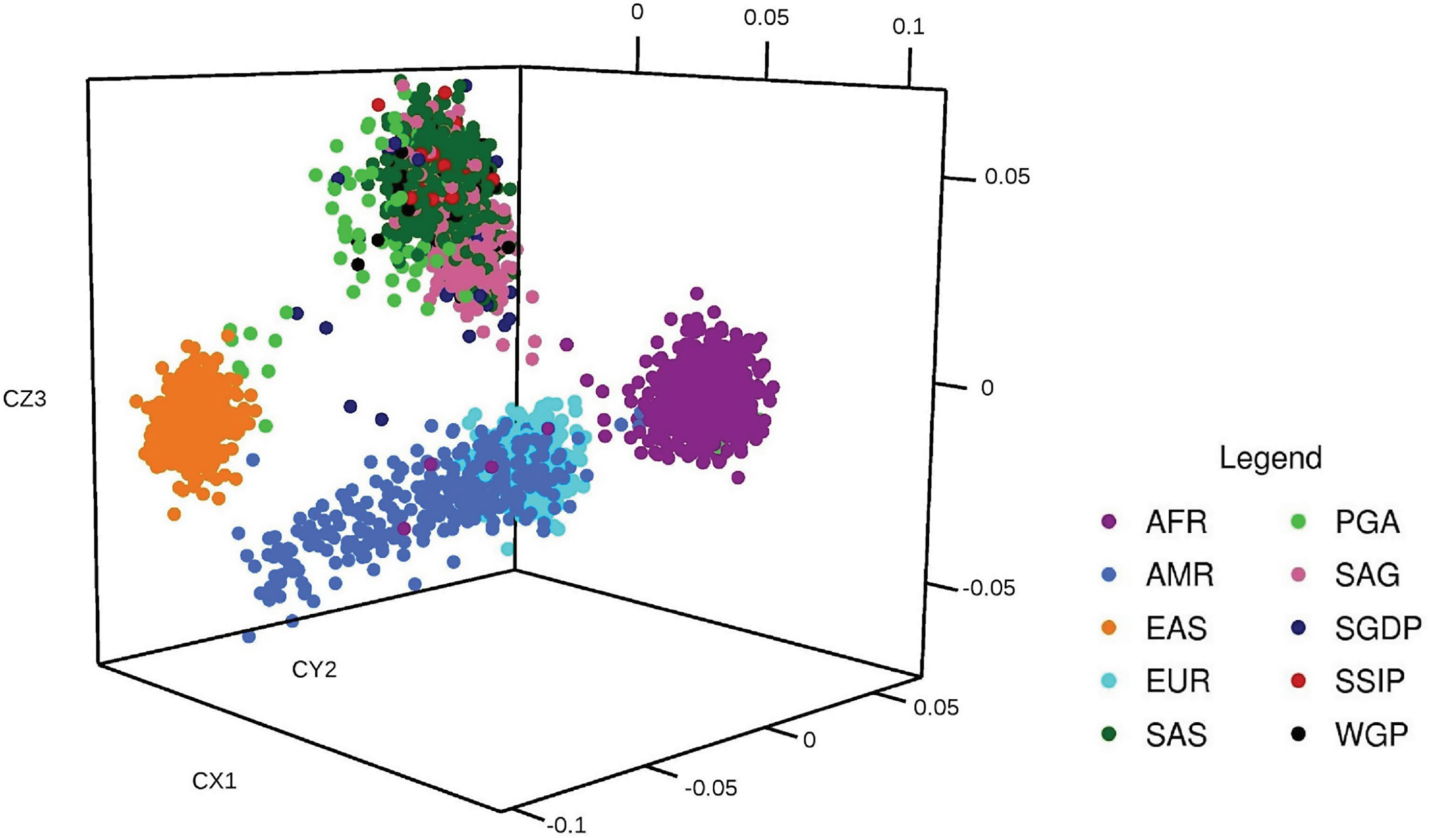
MDS analysis of SAGE: MDS analysis in three-dimensional model revealing the clustering of SAGs into a single cluster. Analysis of SAGE cluster reveals that the samples of SAGE group into a single unique cluster. SAGE cluster is far separated from other groups and as expected SSMP clustered with the other South East Asian cluster. AFR, African; AMR, American; EAS, East Asian; EUR, European; SAS, 1KGP South Asian.

Our analysis successfully recapitulated the interpopulation relationships. The SAS, SSIP, PGA, SGDP, WGP and SAG all grouped together into a single cluster separate from other populations. Five distinct clusters representing different populations could be clearly viewed. The African–American samples of 1KGP AFR grouped with the African cluster. African and Europeans clustered nearby. The North East samples of PGA cohort samples showed affinities to the East Asian samples. The dataset from the Simon’s project was interspersed between the Asian as well as American cluster since a few samples were from Kusunda ethnicity of Nepal and Hazara from Pakistan. Figure 2 shows the multidimensional stratification of all the samples.

### Database interface and features

**Figure 3:**
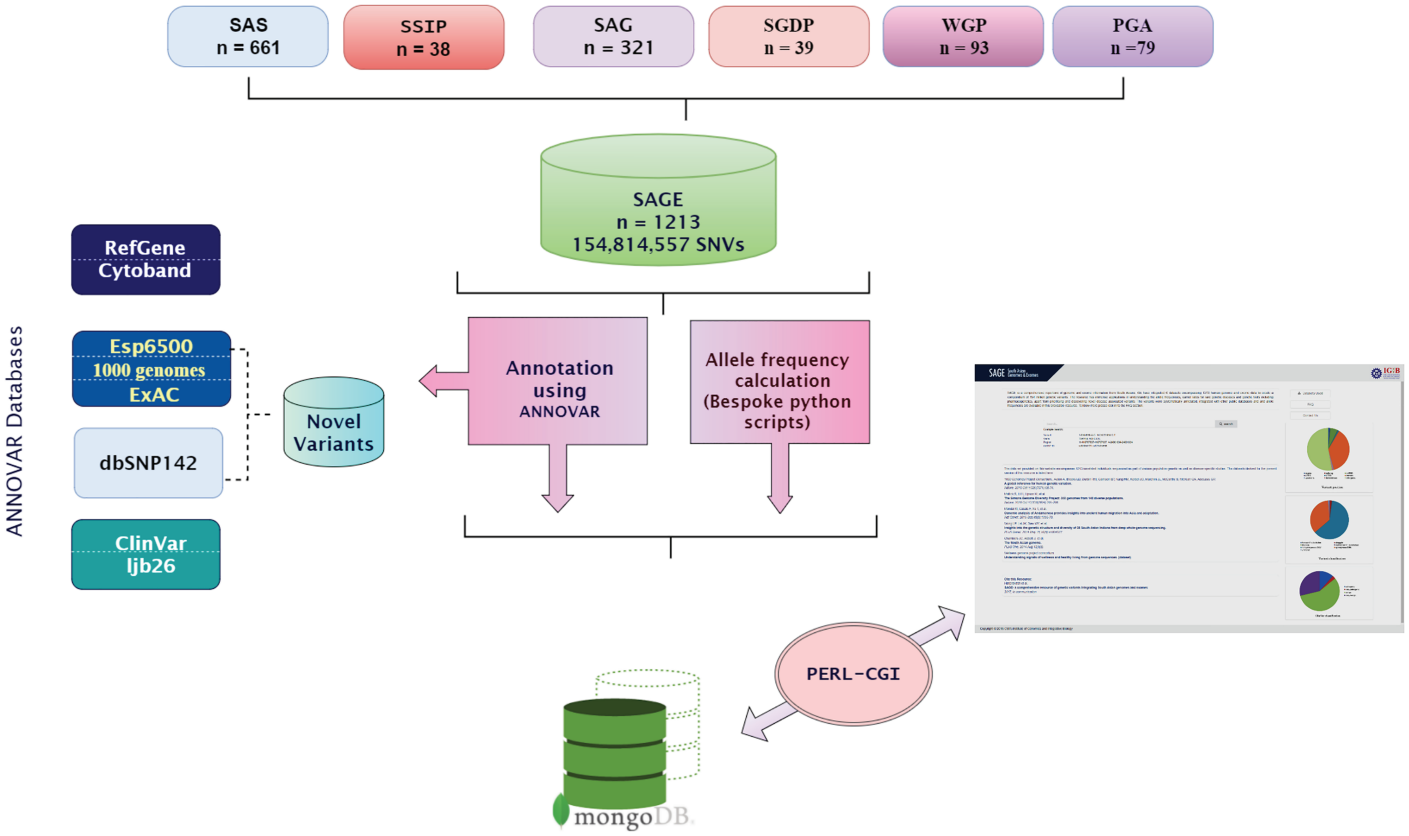
Schematic representation of data integration, annotation and analysis pipeline.

A user friendly web resource was created to enable quick and easy access of variants, allele frequencies, genotype frequencies and relevant annotations. Genetic variant IDs (dbSNP ID), gene names and positions or ranges of genomic positions can be used for query. The interface returns a neatly organized list of links where the user can find more information on the specific variants matching the query condition. The variant page provides details about the variant, its genomic context, population frequency across different populations in the South Asian region as well as across the 1000 genomes, ESP and ExAC datasets and populations. Functional and clinical annotations for each variant have also been precomputed and are available. The variants are also linked out to relevant databases including UCSC Genome Resource, dbSNP and ClinVar. Figure 3 depicts the data integration, annotation and analysis pipeline.

SAGE uses a series of user-friendly interfaces to show results. In order to facilitate accessibility to the database, we provide a downloadable user guide for the database. Briefly, SAGE has four main functionalities: data retrieval, browsing, population specific allele frequency retrieval and variant annotation. The home page introduces the resource and also enlists the data sources of the compendium. Information about the projects, population code, cohort size, description of samples, number of Single Nucleotide Variations (SNV) and number of indels are also provided in the “datasets used” section of the webpage.

**Figure 4:**
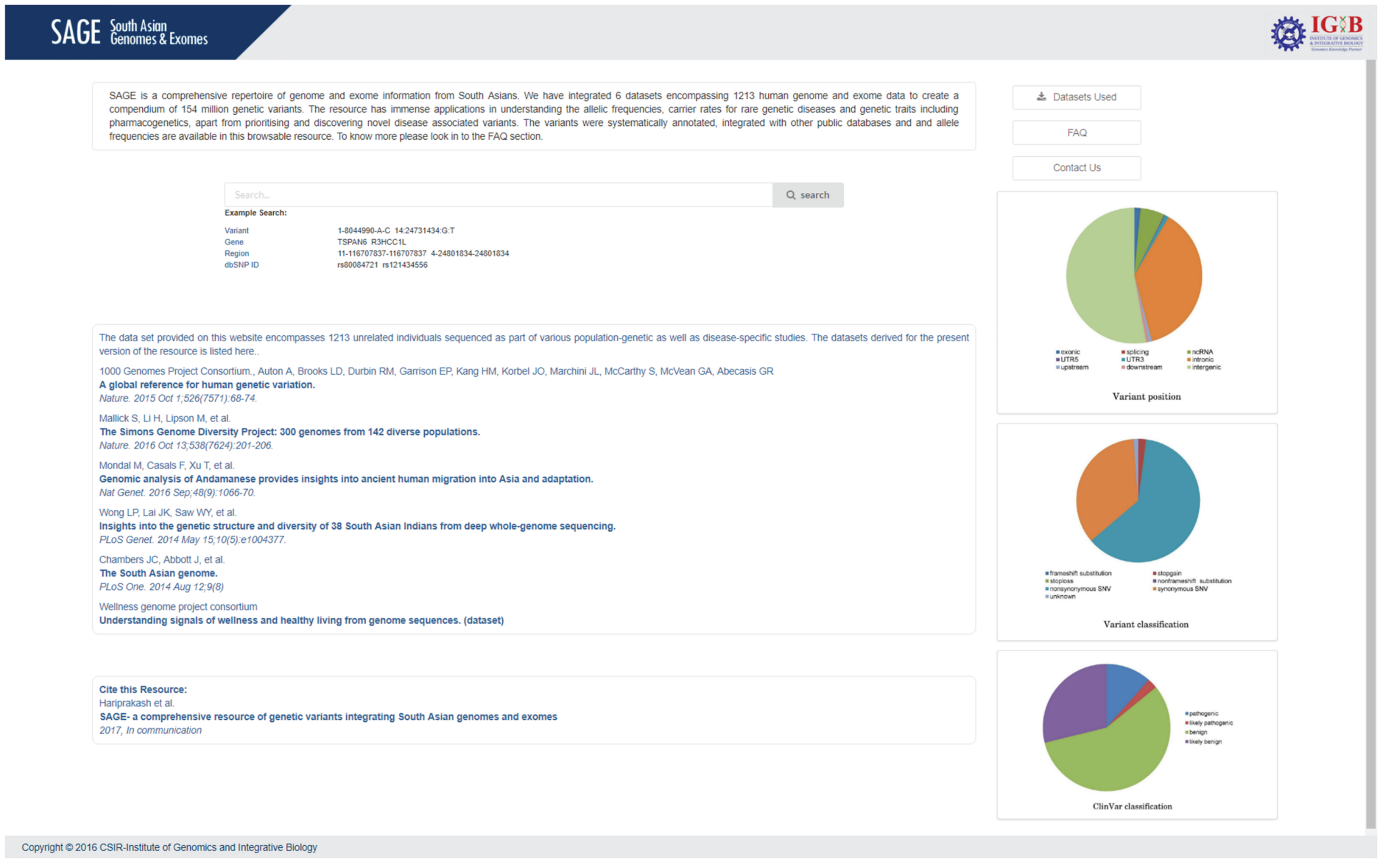
Screenshot of SAGE summarizing the variant search page.

SAGE can be searched by variant, gene name, region or dbSNP ID. Upon searching, an intermediate page containing basic information about the queried variant will be displayed that include variant location, gene name and variant type when the search term is a dbSNP ID or a specific keyword relating to an allele. In case of a gene symbol or a chromosomal region, all the variants in respective gene/region will be enlisted. Each of the entry is provided with a link to expand for the detailed search result. Figure 4 shows the variant search page.

The result page contains two panels: left panel details the annotation of the variant and the right panel provides the carrier frequency of the variant in our datasets as well as in other different population databases.

Variant information includes chromosome number,position, reference allele, alternate Allele (GRCh37/hg19), cytoband, UCSC and dbSNP links for the variant, gene in detail and function of the variant. Each allele has various pathogenicity score and prediction results determined by various computational algorithms to determine the nature of the allele and its clinical importance. A brief summary on each database is also provided for users to understand the importance of each annotation.

**Figure 5:**
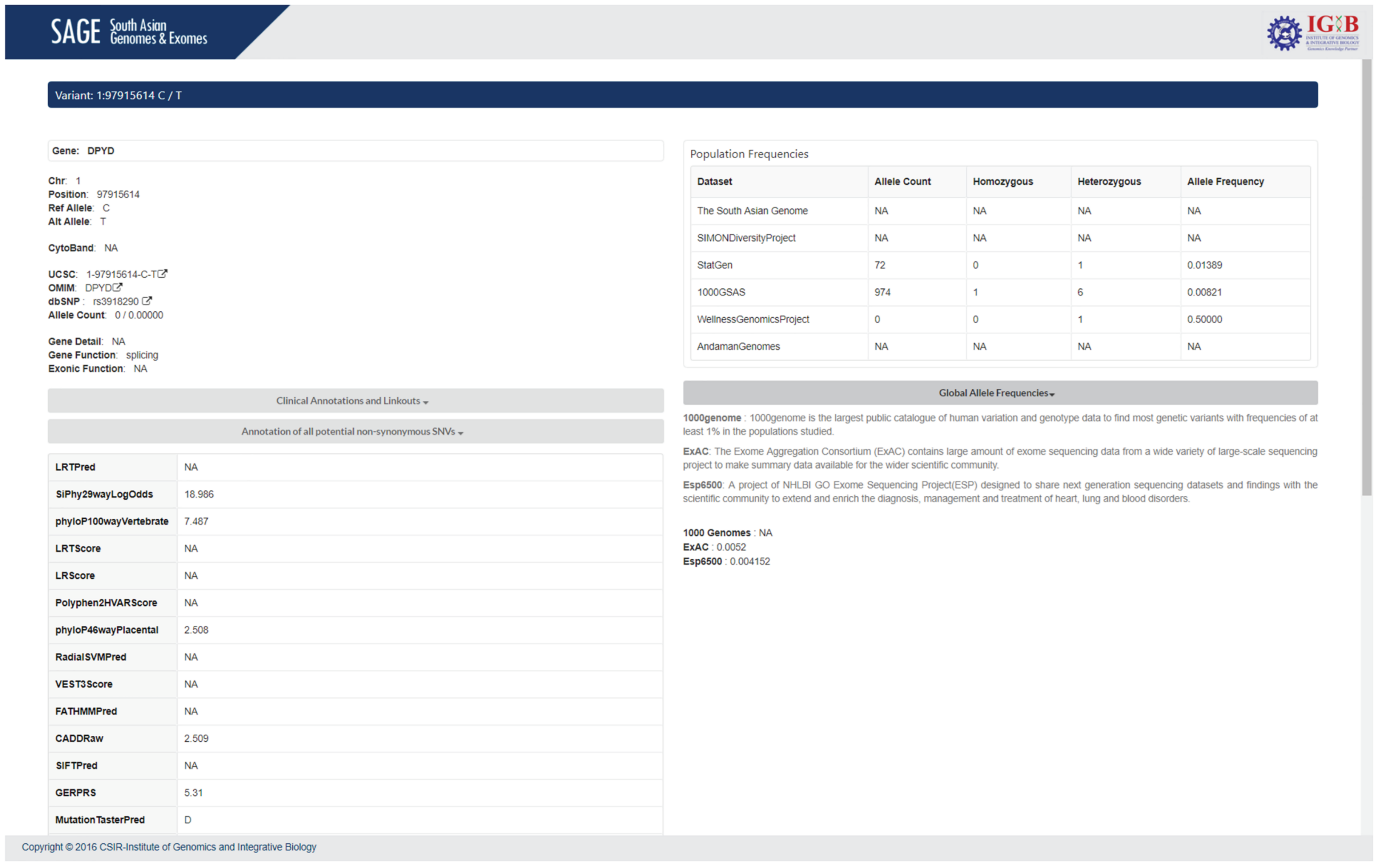
Screenshot of SAGE summarizing the results page.

Clinical annotation and linkouts provides the ClinVar annotation of the variant with link to respective ClinVar page. Annotation of potential non-synonymous SNVs section provides various computational predictions for the pathogenicity of the variant. The Genome Wide Associated Studies (GWAS) Catalogue annotation of the variant is also provided in this section. Population frequencies section provides the carrier frequency rate of the variant of interest in different population datasets. The population details consist of allele count, number of chromosomes, allele frequency, homozygous alleles and heterozygous alleles present for the variant in corresponding cohort. Figure 5 summarizes the result page.

**Table 2:** Description of variants prevalent in South Asia. AD, autosomal dominant; AR, autosomal recessive; condition refers to the type of RP

Variant	Gene	AA change	Condition	Mode of inheritance
rs121909075	TULP1	R420P	RP 14	AR
rs121917744	RPE65	P363T	RP 20	AR
rs62625014	CNGA1	S316F	RP 49	
rs2276717	SLC7A14	G330R	RP 68	AR
rs121912552	IMPDH1	R224P	RP 10	AD
rs121918577	PDE6A	S344R	RP 43	
rs137853005	PROM1	N576TER	RP 41	AR
rs104893770	RHO	F45L	RP 4	AR,AD
rs267607182	ZNF513	C339R	RP 58	AR
rs62635656	CRB1	M1041T	RP 12	AR
rs121909398	CERKL	R257TER	RP 26	
rs104894039	RP9	D170G	RP 9	AD

### Utility of the resource in genetic epidemiology of Mendelian diseases

One of the major utility for an integrated population-specific genomic variant resource would be to enable genetic epidemiology. As a case example, we analyzed the genetic variants associated with RP. It is a Mendelian disorder inherited as autosomal dominant, autosomal recessive and X-linked recessive modes. Ayuso and Millan suggested as many as 87 genes to be associated with RP (31). These genes span a variety of pathways including phototransduction, visual cycle and phagocytosis of rod outer segments or through structure of photoreceptors and ciliary function or involvement in retinal development, mRNA splicing and other functions. More than 40 genes have been correlated with RP, with the majority of them having expression in the photoreceptors or the retinal pigment epithelium (32).

**Figure 6:**
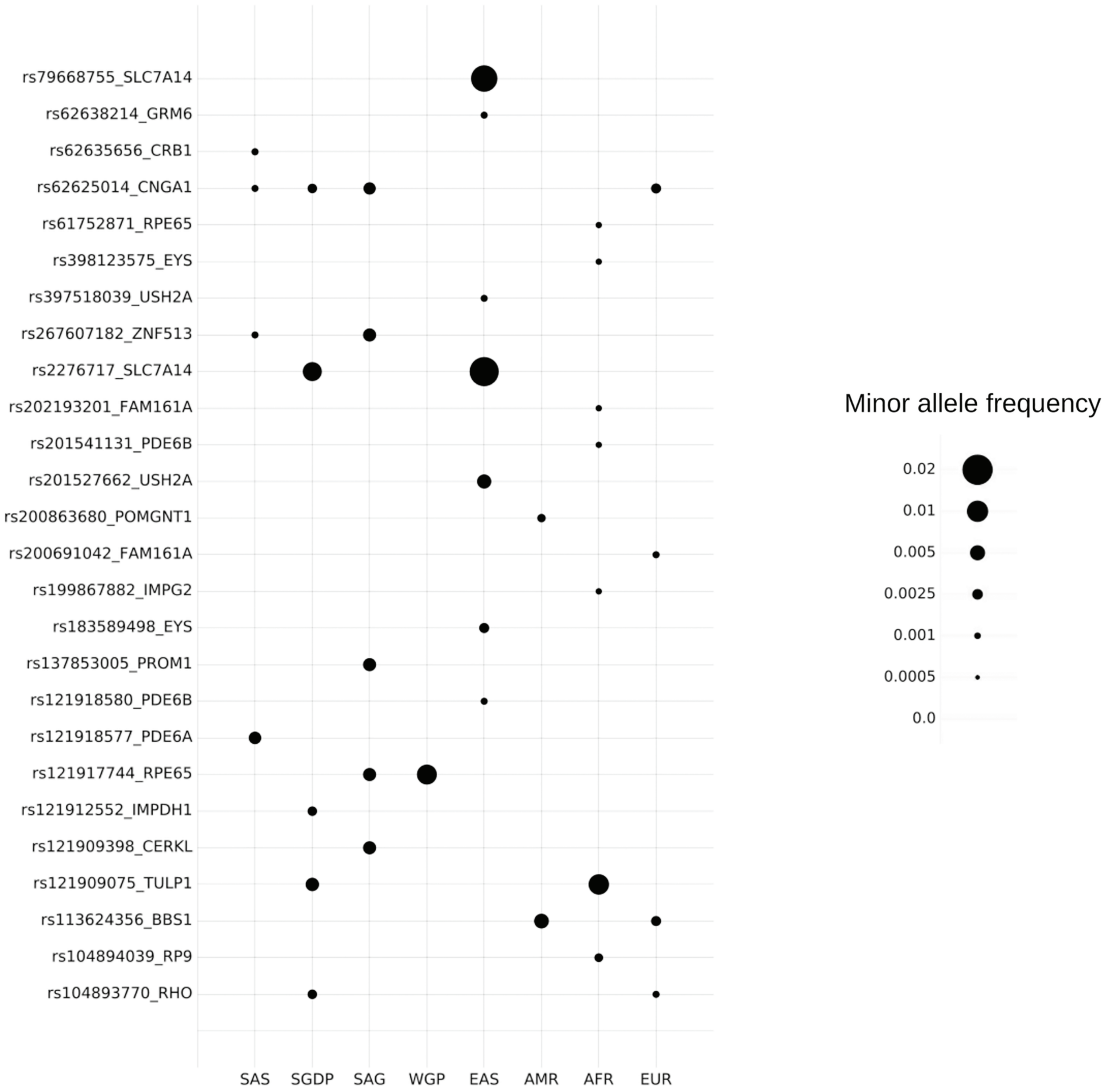
MAF distribution of RP variants. AFR, African; AMR, American; EAS, East Asian; EUR, European; SAS, 1000K South Asian.

The variants annotated as pathogenic in ClinVar were retrieved using bespoke scripts and mapped to the compendium. Our analysis revealed a total of 427 variants associated with RP out of which a total of 27 variants mapped to the SAGE compendium. Table 2 enlists the variants associated with RP and their description. Figure 6 depicts the allelic frequency distribution.

Our analysis revealed the allele frequency for pathogenic variants in the range of 0.001–0.02 in the study population. On average this translates to a cumulative carrier rate of ∼0.01 in the South Asian population. Our analysis revealed the variant rs79668755 in SLC7A14 gene to be prevalent in South East Asian populations with an average frequency of 0.0175 while extremely rare in other populations (34). Most of these variants except for rs104894039 in RP9 gene and rs121912552 in IMPDH1 gene are inherited as an autosomal recessive trait.

## Discussion

Next generation sequencing technology has led to greater insights into the genetic diversity of many Asian populations and has significantly contributed to the understanding of human migrations and admixture. Next generation sequencing data has not only helped in capturing causative variants from massive candidates, it has also been a valuable resource in enlightening the scientific community about a large repertoire of genetic variants.

Disease prevalence, severity and resistance vary substantially in different ethnic groups as a result of both inherited factors and non-inherited causes (13). Occasionally, an SNP or a copy-number variant is relatively common in one population but absent in another (13) making it important to study population specific variation. South Asia is a hub of genetic and ethnic diversity. Cataloging the genetic variants from this region would serve as a blueprint for identification of disease epidemiology, disease associated variants relevant in the population, pharmacogenetic markers that could lead to personalized medicine. The availability of population scale allele frequency distribution would help researchers to compare and analyze clinically relevant variations towards achieving quick translation.

In this study, we have collated whole genome and whole exome datasets from 1213 individuals from seven datasets to create a unique compendium of genetic variants. We uncovered a total of 154 814 557 variants including 69 059 255 novel alleles with each SNV described by 18 functional prediction scores and 20 valuable annotations. More than 0.3 million variants were found to be deleterious in nature by the prediction tools. The database contains information of more than 2000 variants that are classified as pathogenic by ClinVar database. Allele and genotype frequencies for the variants were computed across the populations, which enabled a better understanding of the genetic landscape of various diseases. Allele frequency of variants from other population scale studies like 1000 genomes, ExAC and ESP6500 has also been enlisted for better comparisons. Population stratification analysis of the genomes and exomes remarkably recapitulated the genetic affinities revealed by earlier studies. The South Asian cohort clustered separately, emphasizing its uniqueness and the need for studies specific to them.

For the establishment of utility of the resource in clinical and epidemiological studies, we evaluated the allele frequencies of clinically relevant variants associated with RP. The variants that were prevalent in South Asia have an autosomal recessive mode of inheritance. Mutations in homozygous or in compound heterozygous form has been previously reported in various populations. But there are no previous reports on the allele frequency distribution of these variants in South Asian population and this is the first study reporting it. We observed the variants rs2276717 (SLC7A14) and rs121917744 (RPE65) to have higher occurrence in South Asia. Variant rs121917744 has been reported to cause a non-conservative replacement of the evolutionarily conserved proline-363 by threonine in a consanguineous Indian family by Gu *et al*. (33). This particular family had four individuals with autosomal recessive childhood-onset severe retinal dystrophy. This variant has an allele frequency of 0.0056 in South Asia while the global frequency is 0.00008256 (ExAC). This makes it an important candidate to look at in South Asia. Similarly variant rs2276717 has been reported by Jin *et al.* (34) in the Chinese population. This variant has a higher level of incidence in East Asians also.

This study highlights the need for systematic analysis of clinically relevant genetic variants in the populations and how the availability of a well-curated database would enable in quick and accurate diagnosis, tailoring therapeutics and dosage in specific populations.

The current extent of population sampling would result in more resources. There has been extraordinary progress in genome sequencing technologies since the completion of 1KGP, which has led to an increase in the number and diversity of sequenced genomes. We envision SAGE would be enriched with larger datasets specific to various regions of South Asia and hence could reflect on region specificity and ethnicity within South Asia. To the best of our knowledge, SAGE is the first large-scale comprehensive database for functional predictions and annotations of human whole-genome SNVs from South Asia.

## Supplementary Material

Supplementary Data

## Notes

**Database URL**: http://clingen.igib.res.in/sage
