# Supplementary material for: SAGE: a comprehensive resource of genetic variants integrating South Asian whole genomes and exomes
Source: Database (Oxford). 2018 Sep 13;2018:bay080. doi: 10.1093/database/bay080 (PMC6146123; doi:10.1093/database/bay080)
Supplement: Supplementary Data [file bay080_supp.doc]

| **Variant_annotator** | **No. of deleterious variant** | **Total no. variants** |
| --- | --- | --- |
| FATHMM | 162,429 | 1,038,160 |
| MetaLR | 162,818 | 1,179,557 |
| LRT | 406,392 | 958,584 |
| Mutation Assessor | 39,807 | 1,035,103 |
| Mutation Taster | 659,457 | 1,224,119 |
| Polyphen_hdiv | 413,447 | 1,070,602 |
| Polyphen_hvar | 300,830 | 1,070,602 |
| Radial SVMM | 413,447 | 1,179,557 |
| SIFT | 428,652 | 1,120,478 |

Supplementary Table1: Number of variants annotated as Deleterious by the computational tools.
